# Machine learning-based prediction of longitudinal cognitive decline in early Parkinson’s disease using multimodal features

**DOI:** 10.1038/s41598-023-37644-6

**Published:** 2023-08-14

**Authors:** Hannes Almgren, Milton Camacho, Alexandru Hanganu, Mekale Kibreab, Richard Camicioli, Zahinoor Ismail, Nils D. Forkert, Oury Monchi

**Affiliations:** 1 Department of Clinical Neurosciences, University of Calgary, 2500 University Drive NW, Calgary, AB T2N 1N4 Canada; 2 Hotchkiss Brain Institute, Cumming School of Medicine, University of Calgary, 3330 Hospital Dr NW, Calgary, AB T2N 4N1 Canada; 3 Département de Psychologie, Université de Montréal, Pavillon Marie-Victorin, 90 Vincent d’Indy Ave, Montreal, QC H2V 2S9 Canada; 4 Centre de recherche de l’Institut universitaire de gériatrie de Montréal, 4565 chemin Queen Mary, Montreal, QC H3W 1W5 Canada; 5 Department of Radiology, University of Calgary, 2500 University Drive NW, Calgary, AB T2N 1N4 Canada; 6 Division of Neurology, Department of Medicine, and Neuroscience and Mental Health Institute, University of Alberta, 7-112 Clinical Sciences Building 11350 83rd Avenue, Edmonton, AB T6G 2G3 Canada; 7 Alberta Children’s Hospital Research Institute, Heritage Medical Research Building, University of Calgary, 3330 Hospital Dr. NW, Calgary, AB T2N 4N1 Canada; 8 Département de radiologie, radio-oncologie et médecine nucléaire, Faculté de médecine, Université de Montréal, Pavillon Roger-Gaudry, 2900 Boulevard. Édouard-Montpetit, Montreal, QC H3T 1A4 Canada; 9 Department of Psychiatry, University of Calgary, 3280 Hospital Dr NW, Calgary, AB T2N 4Z6 Canada

**Keywords:** Behavioural genetics, Parkinson's disease, Predictive markers, Cognitive ageing, Cognitive neuroscience

## Abstract

Patients with Parkinson’s Disease (PD) often suffer from cognitive decline. Accurate prediction of cognitive decline is essential for early treatment of at-risk patients. The aim of this study was to develop and evaluate a multimodal machine learning model for the prediction of continuous cognitive decline in patients with early PD. We included 213 PD patients from the Parkinson’s Progression Markers Initiative (PPMI) database. Machine learning was used to predict change in Montreal Cognitive Assessment (MoCA) score using the difference between baseline and 4-years follow-up data as outcome. Input features were categorized into four sets: clinical test scores, cerebrospinal fluid (CSF) biomarkers, brain volumes, and genetic variants. All combinations of input feature sets were added to a basic model, which consisted of demographics and baseline cognition. An iterative scheme using RReliefF-based feature ranking and support vector regression in combination with tenfold cross validation was used to determine the optimal number of predictive features and to evaluate model performance for each combination of input feature sets. Our best performing model consisted of a combination of the basic model, clinical test scores and CSF-based biomarkers. This model had 12 features, which included baseline cognition, CSF phosphorylated tau, CSF total tau, CSF amyloid-beta_1-42_, geriatric depression scale (GDS) scores, and anxiety scores. Interestingly, many of the predictive features in our model have previously been associated with Alzheimer’s disease, showing the importance of assessing Alzheimer’s disease pathology in patients with Parkinson’s disease.

## Introduction

Parkinson's disease (PD) is a movement disorder that affects millions of people worldwide^1^. PD is primarily recognized by its motor symptoms. However, deficits in cognition such as memory impairment are noted even in the early stages of PD^2,3^. In some cases, these deficits can evolve rapidly and often result in dementia^2,4,5^. The ability to identify patients who are predisposed to cognitive decline at early stages of PD could enable early interventions, such as medication^6,7^, cognitive training^8^, neurostimulation^9^, physical exercise interventions^10^, or adaption of living conditions.

The cognitive spectrum in PD can be grouped into three categories: normal cognition (PD-NC), mild cognitive impairment (PD-MCI), and dementia (PD-D)^2^. This categorization is often based on patient and caregiver report, cognitive test scores, and clinical judgement regarding the impact of cognitive decline on function^11,12^. Previous attempts to predict cognitive decline in PD have mainly focused on development of general cognitive impairment^13,14^, progression to dementia^15^, or MCI and dementia^16^. For example, Smith et al*.*^16^ showed that a combination of clinical and brain morphological features performed well (AUC = 0.85) at predicting conversion from healthy cognition to PD-MCI or PD-D. Similarly, Shin et al*.*^15^ showed that models integrating clinical features and cortical thickness outperformed models with only one feature in predicting conversion from PD-MCI to PD-D. Other studies have found that cerebrospinal fluid (CSF) biomarkers are also important predictors of cognitive decline in PD^17,18^. Bäckström et al*.*^17^, for instance, showed that adding CSF biomarkers to a clinical model increased the area under the curve (AUC) from 0.77 to 0.86. Other authors have developed models that include genetics for cognitive decline prediction in PD^19,20^. In a large-scale study, Liu et al*.*^20^ showed that inclusion of GBA gene mutations significantly increased the AUC in the validation set (from 0.827 to 0.854).

Although categorization into MCI or dementia is useful, patients near cut-off thresholds might be misclassified, and sometimes symptom assessments are prone to subjective interpretation. Continuous cognitive scores could give more precise and specific information about a person’s cognitive ability and individual risk^21^. As an alternative to binary classification, Caspell-Garcia et al*.*^22^ attempted to identify biomarkers that can predict decline in continuous cognitive scores using longitudinal regression models. They found that cortical brain morphology (both volume and thickness) of specific brain areas, specific CSF biomarkers, and dopamine deficiency in the dorsal striatum were related to cognitive decline in people with early PD. However, they mainly restricted themselves to linear and logistic regression models, which may not be optimally suited to identify complex non-linear relationships in the data. More advanced machine learning methods enable the detection of patterns in high-dimensional data, and can be used to predict future outcomes in patients^23^. To date, machine learning has shown great promise for many precision medicine applications^24^. With respect to PD, machine learning has been used for predicting cognitive decline in PD after deep brain stimulation^25^, and conversion from PD-MCI to PD-D^15^.

The aim of this study was to develop, evaluate, and compare unimodal and multimodal machine learning models for the prediction of cognitive decline, as measured by change in continuous cognitive scores, in patients suffering from early PD using demographic, clinical, imaging, genetic, and CSF data.

## Results

### Participants characteristics

After applying the study exclusion and inclusion criteria, our final subject sample included 213 subjects from 23 sites (see Supplementary Fig. 1 for details on subject exclusion). Baseline demographic and clinical data for our subject sample are shown in Table 1. The average MoCA change over 4 years for our participants was -0.45, the average age was 61 years, 34.3% were female, and the median duration of education was 16 years. Median duration of disease was 4.4 months, the average MDS-UPDRS part III score was 20.0, and the average MoCA score at baseline was 27.

**Table 1:** Baseline demographic and clinical data. Abbreviations: std = standard deviation; IQR = interquartile range. ^a^These scores are off-medication.

Variable	Sample characteristics
**Number of subjects**	**213**
**Age** (Years)	
Mean (std; min–max)	**61.28** (9.9; 33.5–84.9)
**Sex**	
Total Female (percentage)	**73** (34.3%)
Total Male (percentage)	**140** (65.7%)
**Years of education**	
Median (IQR; min–max)	**16** (4; 8–26)
**Duration of disease** (months)	
Median (IQR; min–max)	**4.4** (5.0; 0.4–35.8)
**MoCA score at baseline**	
Mean (std; min–max)	**27** (2.2; 17–30)
**MoCA change over 4 years**	
Mean (std; min–max)	− **0.45** (3.2; **− **16–7)
**MDS-UPDRS part III score**	
Mean (std; min–max)	**20.0** (8.1; 4–41)
**Hoehn and Yahr staging**^a^	
Total Stage 1	**100** (46.9%)
Total Stage 2	**111** (52.1%)
Total stage 3	**2** (1.0%)

### Machine learning model results

**Figure 1:**
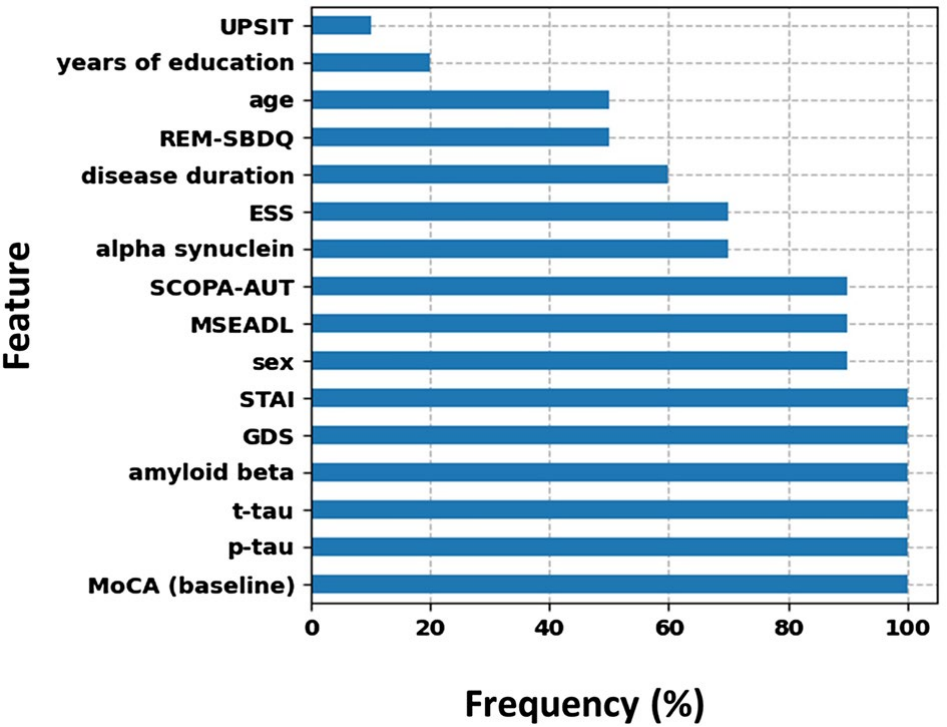
Frequencies across all 10 left-out folds (in percentages) for each feature that was selected as part of the 12 highest ranked features in at least one fold. *Abbreviations:* UPSIT = University of Pennsylvania Smell Identification Test, REM-SBDQ = REM Sleep Behavior Disorder Questionnaire, ESS = Epworth Sleepiness Scale, SCOPA-AUT = Scales for Outcomes in Parkinson’s Disease—Autonomic Dysfunction, MSEADL = Modified Schwab & England Activities of Daily Living Score, STAI = State-Trait Anxiety Inventory (total score), GDS = Geriatric Depression Scale, t-tau = CSF total tau, p-tau = CSF phosphorylated tau, amyloid beta = CSF amyloid beta.

The best model for the prediction of cognitive decline in PD consisted of clinical test scores and CSF-based biomarkers, in addition to demographics and baseline cognition. The correlation between predicted and observed scores was 0.44. The best model within this combination of feature sets included 12 predictors (ranked and selected with RReliefF). Figure 1 shows the percentage of folds for which each feature was selected. Baseline MoCA, CSF phosphorylated tau, CSF total tau, CSF amyloid beta, geriatric depression scale (GDS) scores, and state-trait anxiety inventory (STAI) total scores were consistently part of the predictor set for each cross-validation iteration. Moreover, sex, activities of daily living scores, and autonomic function scores were selected in 90% of the iterations. Finally, CSF alpha-synuclein and Epworth sleepiness scale (ESS) scores were part of the model in seven out of ten iterations. Supplementary Fig. 2 shows the scatterplot between observed and predicted MoCA change.

### Univariate associations

Statistically significant positive associations with change in MoCA score (indicating that higher scores are related to less cognitive decline) were found for CSF amyloid-beta (t = 2.13; *p* = 0.018), while statistically significant negative relationships (indicating that higher scores are related to more cognitive decline) were found for baseline MoCA (t = − 4.26; *p* = 0.00004), CSF total tau (t = − 1.66; *p* = 0.049), total STAI scores (t = − 1.73; *p* = 0.042), and autonomic dysfunction (t = − 2.57; *p* = 0.005). The effect of CSF phosphorylated tau on MoCA change was close to significance (t = − 1.64; *p* = 0.052). Results for all features are reported in Supplementary Table 1.

## Discussion

In this study we developed, compared, and evaluated unimodal and multimodal machine learning models to predict cognitive decline in Parkinson’s disease (PD) over four years using a wide range of features from different modalities, including CSF biomarkers, genetics, regional brain volumes, and clinical test scores. Our best performing model included CSF markers and clinical test scores, in addition to simple demographics and baseline cognition. Commonly selected variables included CSF phosphorylated tau, CSF total tau, CSF amyloid-beta, geriatric depression scores, anxiety scores, sex, functional independence scores, autonomic dysfunction scores, daytime sleepiness, and CSF alpha-synuclein. The Pearson correlation between predicted and actual MoCA decline was 0.44, while the mean absolute error of the prediction was 2.11 in units of change in total MoCA score.

The best performing model included CSF-based biomarkers and clinical test scores, but no brain volumes or genetics data. The importance of CSF biomarkers is in line with the studies of Bäckström et al*.*^17^ and Siderowf et al*.*^18^, who identified CSF amyloid-beta_42_ as a predictor of cognitive decline in PD. The importance of clinical test scores has been shown by both Shin et al*.*^15^ and Smith et al*.*^16^, who found that a combination of clinical test scores and brain structure is better at predicting conversion to dementia and MCI in PD compared to each set separately. The present study confirms that clinical test scores and CSF biomarkers are essential for the prediction of cognitive decline in PD. In contrast, brain volumes and genetics were not part of the best performing model in our study. One reason could be that genetics and CSF markers are associated (see, e.g., Kanekiyo et al*.*^26^ for amyloid-beta and APOE ε4) which could make either of them redundant as a predictor. In contrast to genetics, CSF biomarkers measure the actual presence of protein pathology which might make them relatively more powerful in detecting and predicting cognitive decline. Similarly, hippocampal volume is known to be related to amyloid-beta pathology^27,28^, which could also make either of them a redundant feature. CSF biomarkers are likely a closer measure of underlying pathology compared to brain volumes that may not be as precise to determine, which could make them relatively more important for prediction of cognitive decline.

Regarding specific CSF biomarkers we found that phosphorylated tau, total tau, and amyloid beta_1-42_ were consistently selected predictors of cognitive decline. CSF amyloid-beta_42_ has been found to be an important predictor for cognitive decline in PD in other studies^17,18^. Similarly, the results on phosphorylated and total tau are in line with other studies showing that tau pathology is related to dementia in PD^29,30^. Compta et al*.*^29^, for instance, showed that Braak tau stages are significantly greater in PD with dementia compared to PD without dementia. This study also showed a negative correlation between Braak tau stages and cognitive performance in PD at the end of life. However, some studies did not identify CSF tau as a predictive feature of cognitive decline in PD^18^. CSF alpha-synuclein was also identified as a predictive feature in the majority of our cross-validation folds, which is in line with other research on dementia in PD showing an association between alpha-synuclein and Parkinson’s disease dementia^30^. CSF alpha-synuclein was, however, a lower ranked predictor compared to the other CSF biomarkers.

Most of the selected clinical features in our study have been previously identified in studies assessing correlates and predictors of cognitive decline in PD. Excessive daytime sleepiness has been found to be related to global cognition and specific cognitive functions, both cross-sectionally and longitudinally^31^. Moreover, depression has been identified as an important predictor of cognitive decline in PD^20^ and has been shown to be higher in converters to PD-MCI and PD-D compared to non-converters^16^. In the latter study, autonomic dysfunction was also found to be part of the optimal feature set^16^. Instrumental activities of daily living have been previously found to be related to executive function, attention deficits, and MCI^32,33^. Many of the predictive properties of clinical test scores have been related to impaired functional properties of the brain, such as altered neurotransmitter release and re-uptake. For instance, excessive daytime sleepiness has been linked to abnormal dopamine transporter binding in the caudate nucleus^34^. Acetylcholinesterase density was found to be decreased in the small intestine and pancreas of PD patients, potentially due to underlying issues with autonomic function (*e.g.,* constipation;^35^). Instrumental activities of daily living have been shown to be sensitive to cholinesterase inhibitors^36^, suggesting a cholinergic underpinning. Finally, depression and anxiety have been associated with altered dopaminergic, serotonergic, and noradrenergic mechanisms^37^. In the case of depression, some negative effects of motivation on cognitive test performance might also play a role^38^. Interestingly, pure motor scores (*e.g.,* MDS-UPDRS part III) were not found to be part of the predictive feature set for cognitive decline in neither Smith et al*.*^16^ nor in the present study, although UPDRS Part III was significantly higher in converters than in non-converters in Smith et al*.*^16^ and was part of the predictive set in Bäckström et al*.*^17^.

Our univariate analyses showed that CSF beta-amyloid was significantly associated with less cognitive decline, whereas higher baseline MoCA, CSF total tau, anxiety, and autonomic dysfunction were significantly related to more cognitive decline. Most of these results are in line with previous research^2,16,27,28^. Interestingly, we found a significant negative correlation between baseline MoCA and cognitive decline, indicating that participants with higher cognitive abilities showed the most cognitive decline over time. Several explanations are possible for this finding. It is possible that context-effects partly explain the negative relationship between baseline MoCA and change in MoCA scores. The presence of anxiety because of the new environment, or unfamiliarity with the test procedure, could have made some patients score lower at baseline, while in reality their baseline cognitive performance could have been higher than the test score indicated. We indeed found a slight negative correlation (r = -0.06) between state anxiety and baseline MoCA score (not reported in results). However, given the small magnitude of the correlation, this likely does not fully explain our observation. Regression-to-the-mean could also explain the predictive effect of baseline cognition^21^.

In summary, many of the predictive features of cognitive decline in PD identified in this work, such as CSF amyloid-beta, and tau pathology, have previously been associated with Alzheimer’s disease^39–41^. Thus, our study adds to the body of evidence of an important pathological overlap between cognitive decline in PD and AD^42–44^.

### Limitations and future avenues

Our study sample focused on early PD, in which cognitive decline was found to be quite small in general. Moreover, test–retest effects could have led to inflated MoCA scores at later timepoints^45^. Predictive performance and selected features in our models might be different in studies without annual repetition of the same task (PPMI assesses MoCA yearly). Future studies could focus on datasets with less repetition of cognitive tests, on tests with less practice effects, or could use datasets that show more cognitive decline, such as datasets that include participants at later stages of PD. Moreover, it is also important to focus on more precise measures of cognitive performance in future studies, possibly by combining multiple extensive cognitive tests. Multimodal imaging (*e.g.,* including diffusion-weighted imaging) could also lead to improved predictive performance^46^. Deep learning algorithms, such as convolutional neural networks, also have the potential to improve predictive performance, provided large-scale data is available^47,48^. Finally, the generalizability of our results would ideally be assessed with an independent out-of-sample test set.

## Conclusion

Our best machine learning model for cognitive decline in de novo Parkinson’s disease (PD) consisted of CSF biomarkers and clinical test scores, in addition to basic demographics and baseline cognition. Pearson correlation between predicted and observed MoCA change was 0.44. The predictive features in our model included features that have previously been linked to Alzheimer’s disease (AD), such as CSF amyloid-beta and tau concentration. Thus, our study adds to the body of evidence showing an important overlap between features related to cognitive decline in PD and AD.

## Materials and methods

### Dataset and subjects

The data were obtained from the Parkinson’s Progression Markers Initiative database (PPMI; https://www.ppmi-info.org/). PPMI is an observational multi-center study that collects different types of longitudinal data (*e.g.,* cognitive, clinical, T1-weighted MRI) from de novo PD patients without dementia and control subjects^49^. Each PPMI recruiting center received written informed consent from all the participants in accordance with the declaration of Helsinki. Each center also received ethics approval from their local ethics board. We do not have the name of the local ethics committees, as the dataset was acquired by PPMI. The present study was also approved by the Conjoint Health Research Ethics Board at the University of Calgary. We only included PD patients that met the following criteria: (1) a diagnosis of PD at the start of the study, (2) availability of a T1-weighted MRI scan and all features at baseline used in the present study, and (3) completeness of a Montreal Cognitive Assessment (MoCA) test score at baseline and approximately 4 years post-baseline (more precisely, between 3.5 and 4.5 years post-baseline). Supplementary Fig. 1 shows a flowchart of subject selection and exclusion.

### Cognitive assessment

Global cognitive ability was assessed at baseline and at follow-up using the Montreal Cognitive Assessment (MoCA). MoCA is a brief assessment that takes approximately ten minutes to complete, developed and used to detect MCI^50^. The primary outcome of our study was the difference in the total score between follow-up MoCA (acquired 3.5 to 4.5 years after baseline) and baseline MoCA scores. Here, negative values reflect a decline in test scores, while positive values reflect an improvement in test scores.

### MRI acquisition and processing

#### MRI acquisition

T1-weighted MRI images were acquired with scanners and scan sequences differing between the 23 PPMI acquisition sites. MRI scanner vendors used for the acquisition of the data in the present study included Siemens (134 subjects), GE (56 subjects), and Phillips (23 subjects). Magnetic field strength was either 3.0 T (119 subjects) or 1.5 T (94 subjects). Slice thickness varied between 1.0 and 2.0 mm, and almost all images were acquired in the sagittal orientation (92% of subjects).

#### FreeSurfer processing

T1-weighted images were processed using FreeSurfer (version 7.1.1), which included removal of non-brain tissue, intensity normalization, tessellation of the grey/white matter borders, segmentation of subcortical structures, as well as cortical parcellation. Cortical volume was estimated for each of the 62 regions (31 per hemisphere) that are part of the Desikan–Killiany–Tourville (DKT) brain region atlas^51^ using standard automatic procedures (https://surfer.nmr.mgh.harvard.edu/fswiki/recon-all)^52–55^. Subcortical volumetry was estimated for 18 regions (see, Table 2)^56–59^. Both cortical and subcortical volumetry was corrected for (*i.e.,* divided by) total intracranial volume. Parcellation and segmentation results were visually checked and data showing mis-registration or mis-segmentations affecting the morphological analyses were excluded, which involved 10 subjects (for details see Supplementary Fig. 1).

**Table 2:** The complete set of input features used in this study. ^a^Bilateral regions were included for amygdala, hippocampus, thalamus, caudate nucleus, putamen, pallidum, and accumbens area. *Abbreviations:* APOE = apolipoprotein E, MAPT = microtubule associated protein tau, GBA = glucosylceramidase beta 1, BDNF = Brain Derived Neurotrophic Factor, COMT = Catechol-O-methyltransferase, LRRK2 = Leucine-rich repeat kinase 2, SNCA = synuclein alpha, CSF = cerebrospinal fluid.

Features
**Demographics**
Age, sex, years of education, handedness, disease duration
**Cognition**
Baseline MoCA score
**Clinical Tests**
Epworth Sleepiness Scale (ESS)
REM Sleep Behavior Disorder Questionnaire (REM-SBDQ)
University of Pennsylvania Smell Identification Test (UPSIT)
MDS-Unified Parkinson's Disease Rating Scale (MDS-UPDRS) Part III
Modified Schwab & England Activities of Daily Living Score (MSEADL)
Scales for Outcomes in Parkinson’s Disease—Autonomic Dysfunction (SCOPA-AUT)
State-Trait Anxiety Inventory (STAI)
Geriatric Depression Scale (GDS)
**CSF biomarkers**
Amyloid beta_1-42_
Total tau
Phosphorylated tau
Alpha-synuclein
**Cortical regional volumes** (all bilateral)
**Frontal:** superior frontal gyrus, middle frontal gyrus (rostral, caudal), inferior frontal gyrus (pars opercularis, triangularis, orbitalis), orbitofrontal cortex (lateral, medial), precentral gyrus, paracentral lobule
**Parietal:** superior parietal lobule, inferior parietal lobule, precuneus, supramarginal gyrus, postcentral gyrus
**Occipital:** cuneus, lateral occipital cortex, pericalcarine cortex, lingual gyrus
**Temporal:** entorhinal cortex, parahippocampal gyrus, fusiform gyrus, inferior temporal gyrus, middle temporal gyrus, superior temporal gyrus, transverse temporal gyrus
**Cingulate:** anterior cingulate cortex (caudal and rostral), posterior cingulate cortex, isthmus of cingulate gyrus
**Insula**
**Subcortical volumes**^a^
Amygdala, hippocampus, thalamus, caudate nucleus, putamen, pallidum, accumbens area, medulla, pons, superior cerebellar peduncle (SCP), midbrain
**Total brain volume**
**Genetic variants**
APOE ε4
MAPT (rs17649553)
GBA (N370S)
BDNF (rs6265)
COMT (val158met)
LRRK2 (G2019S)
SNCA (rs356181)
SNCA (rs3910105)

### Feature set

The full set of features extracted and available for our study is shown in Table 2. The features included five demographic variables, baseline MoCA score, scores on eight clinical tests, four CSF biomarkers, volumes of 18 subcortical and 62 cortical regions (divided by total intracranial volume), total brain volume, and eight genetics variants. The five demographics were age, sex, years of education, handedness, and disease duration. Clinical tests included Epworth Sleepiness Scale (ESS), REM Sleep Behavior Disorder Questionnaire (REM-SBDQ), the University of Pennsylvania Smell Identification Test (UPSIT), MDS-UPDRS Part III, Modified Schwab & England Activities of Daily Living Score (MSEADL), Scales for Outcomes in Parkinson’s Disease-Autonomic Dysfunction (total score; SCOPA-AUT), State-Trait Anxiety Inventory (total score; STAI), and Geriatric Depression Scale (GDS). The four CSF biomarkers were amyloid-beta_1-42_, total tau, phosphorylated tau, and alpha-synuclein. We included genes that have been associated with cognitive decline in previous studies^60^. The genetic mutations we included as features were: APOE ε4, MAPT (rs17649553), GBA (N370S), BDNF (rs6265), COMT (val158met), LRRK2 (G2019S), SNCA (rs356181 and rs3910105). The input features were categorized into four sets consisting of clinical test scores, CSF-based markers, brain volumetry, and genetics, as well as a basic feature set including demographics and baseline cognition.

The outcome of our machine learning model was the difference in the total score between the follow-up and the baseline MoCA test.

### Machine learning pipeline

**Figure 2:**
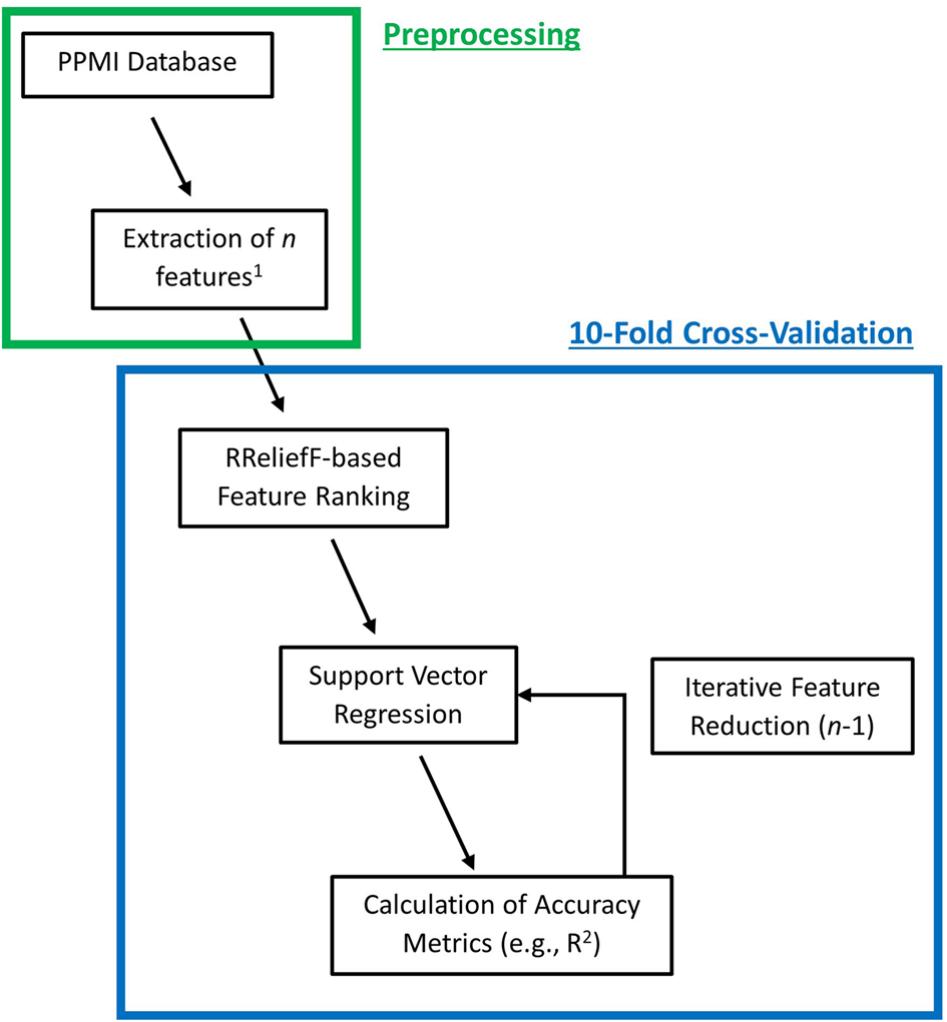
Illustration of the machine learning pipeline. Features (*e.g.,* brain volume, genetics) were extracted from the PPMI database. RReliefF based feature ranking and support vector regression with a polynomial kernel were applied to each fold in the tenfold cross-validation. This procedure was repeated for iteratively reduced features sets (lowest ranked features were removed one at a time) until only one feature was left. The feature set with highest R^2^ across folds was considered the best model.

The machine learning pipeline used in this work consisted of an initial feature ranking followed by training of a support vector regression (SVR) model with a polynomial kernel (see Fig. 2). The RReliefF algorithm, which is an extension of the ReliefF algorithm to regression problems^61^, was used for feature ranking. Briefly described, this method assigns a weight to each feature based on the extent to which its values can differentiate between different outcomes and based on the extent to which different features contain unique information^62^. Based on the RRelieFF feature ranking, the machine learning model was iteratively trained with reduced feature sets. More precisely, the least important feature was iteratively removed from the feature list used for training and evaluation of our machine learning models. Starting with the full set of ranked features, this procedure was repeated until only a single feature was left for training of each machine learning model. Feature ranking was performed individually for each machine learning model and was part of the cross-validation (*i.e.,* feature ranking was performed using the training set only) to prevent a potential double dipping.

Support vector regression models were trained and evaluated based on the ranked and selected features for the prediction of cognitive decline. Accuracy of the machine learning model for each subset of the features was assessed using tenfold cross validation. All combinations of the four feature sets (*i.e.,* clinical test scores, CSF biomarkers, genetics, and brain volumes) were added to the basic model (consisting of demographics and baseline cognition) to form the input feature sets that were compared. The optimal combination of input feature sets and the optimal number of features were determined using the R^2^ metric.

### Statistical analyses

We explored univariate linear relationships between the predictors in our machine learning model and change in MoCA score. Permutation-tests for linear models with 50,000 permutations were used to determine statistical significance. Each linear model had one feature as predictor and change in MoCA score as outcome. We used one-sided tests for effects for which we had a prior expectation of the direction, which was *positive* for activities of daily living (*i.e.,* more independence would implicate less decline) and CSF amyloid-beta, and *negative* for CSF phosphorylated and total tau, geriatric depression scores, anxiety scores, daytime sleepiness scores, and autonomic dysfunction. Two-sided tests were used for features for which we did not have a prior expectation, which were baseline MoCA, CSF alpha-synuclein, and sex. A *p*-value < 0.05 was considered statistically significant.

### Supplementary Information

Supplementary Information.

## Data Availability

Data used in the preparation of this article were obtained from the Parkinson’s Progression Markers Initiative (PPMI) database (https://www.ppmi-info.org/access-data-specimens/download-data). For up-to-date information on the study, visit www.ppmi-info.org.
