# Supplementary material for: Machine learning-based prediction of longitudinal cognitive decline in early Parkinson’s disease using multimodal features
Source: Sci Rep. 2023 Aug 14;13:13193. doi: 10.1038/s41598-023-37644-6 (PMC10425414; doi:10.1038/s41598-023-37644-6)
Supplement: Supplementary file 1 — Supplementary Information. [file 41598_2023_37644_MOESM1_ESM.pdf]

Supplementary Material

Machine learning-based prediction of longitudinal cognitive decline in early Parkinson's disease using multimodal features

Hannes Almgren, Milton Camacho, Alexandru Hanganu, Mekale Kibreab, Richard Camicioli, Zahinoor Ismail, Nils D. Forkert, Oury Monchi

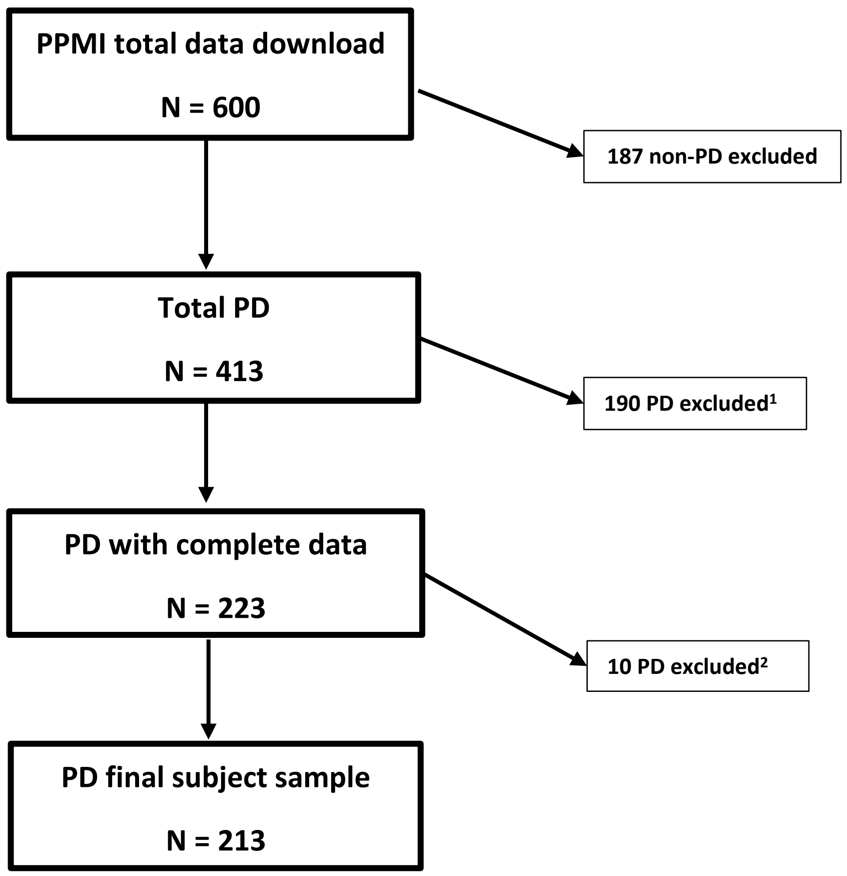

Supplementary Figure 1. Flowchart for subject selection and exclusion. [1]Excluded because data was missing (e.g., follow-up MoCA score between 3.5 and 4.5 years was unavailable or some of the genetic data was missing), [2]Excluded because of issues with FreeSurfer estimation or misregistration of the atlas.

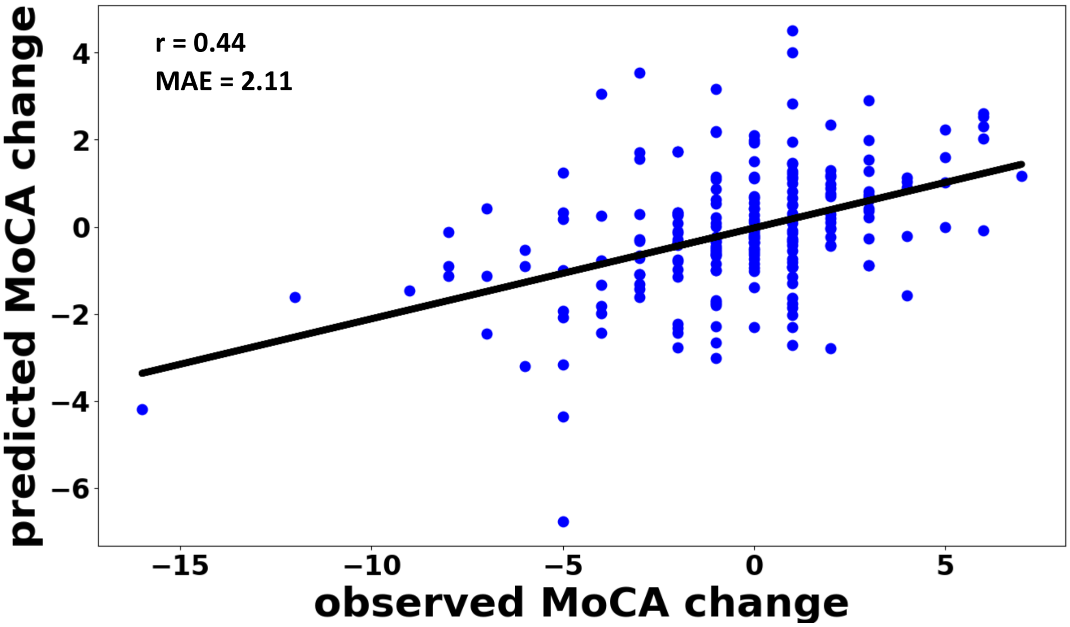

Supplementary Figure 2. Scatterplot between observed and predicted change in MoCA scores. The figure shows the values (blue circles), Pearson correlation (top left corner), mean absolute error (MAE; top left corner), and best linear fit (black line). The formula for the best linear fit line is: y = 0.21x - 0.03, with y = predicted change, and x = observed change. *Abbreviations:* MoCA = Montreal Cognitive Assessment, r = Pearson correlation, MAE = mean absolute error.

**Feature**	**T-value**	**p-value**
Baseline cognition	-4.26	0.00004
CSF p-tau	-1.64	0.052
CSF t-tau	-1.66	0.049
CSF abeta	2.13	0.018
Geriatric depression score	-0.46	0.32
STAI total score	-1.73	0.042
Sex[1]	-0.79	0.217
Activities of daily living	-0.68	0.75
Autonomic dysfunction	-2.57	0.005
CSF α-syn	0.20	0.84
Daytime sleepiness	-0.79	0.22

Supplementary Table 1. List of univariate associations of the change in MoCA scores and features that were part of our model in at least 70% of folds. [1]male = 0, female = 1. *Abbreviations:* CSF = cerebrospinal fluid, p-tau = phosphorylated tau, t-tau = total tau, abeta = amyloid $beta_{1-42}$, α-syn = alpha-synuclein, STAI = State-Trait Anxiety Inventory.
